# Safety and Protective Efficacy of a Candidate Vector-Based Vaccine for Bovine Tuberculosis

**DOI:** 10.3390/vaccines11071199

**Published:** 2023-07-04

**Authors:** Zhandos Abay, Ainur Nurpeisova, Kamshat Shorayeva, Sandugash Sadikaliyeva, Bolat Yespembetov, Nazym Syrym, Makhpal Sarmykova, Kuanysh Jekebekov, Ruslan Abitayev, Gaukhar Tokkarina, Elina Kalimolda, Zharkinay Absatova, Sabina Moldagulova, Han Sang Yoo, Markhabat Kassenov, Kunsulu Zakarya, Yergali Abduraimov

**Affiliations:** 1 Research Institute for Biological Safety Problems, Guardeyskiy uts 080409, Kazakhstan; abaizh097@mail.ru (Z.A.);; 2 Department of Biotechnology, Al-Farabi Kazakh National University, Almaty 050040, Kazakhstan; 3 College of Veterinary Medicine, Seoul National University, Seoul 08826, Republic of Korea

**Keywords:** bovine tuberculosis, influenza vector, safety, protective efficacy, histology

## Abstract

This study presents the results of a survey of the safety and protective efficacy of a candidate vector-based vaccine for bovine tuberculosis, using an influenza vector with the NS1 mutation and expressing M. bovis protective antigens ESAT-6 and TB10.4. We vaccinated Balb/c outbred mice two times at 21 days apart. Our experimental design includes mice immunised with the candidate vaccine with or without adjuvant 15% Montanide Gel. The candidate vaccine’s safety was determined by biometric analysis, and protective efficacy was assessed by bacteriological and histological experiments following a virulent M. bovis-8 strain challenge. Our data indicated that the adjuvant-free version of the vaccine ensured complete protection from the M. bovis-8 infection in mice.

## 1. Introduction

Bovine tuberculosis is a chronic infectious disease caused by Mycobacterium bovis (M. bovis) that affects domestic and wild animal species [1,2,3]. The disease is reported in cattle worldwide despite prophylactic activities involving regular PPD testing and culling the infected animals to reduce transmission and disease-induced morbidity [4].

Vaccination prevents infection in humans and animals. However, immunisation with inactivated and live attenuated vaccines has disadvantages such as post-vaccination allergic reactions and short-lived acquired immunity [5]. An alternative to traditional vaccines is a vector-based approach that delivers vaccine antigens-associated gene fragments. A vector vaccine provides a robust stimulation with high specificity immune response associated with safety to the host [5].

Influenza vector-based vaccines are safe against infectious diseases in humans and animals. The influenza vector is characterised by 50% truncation in the translated region of the NS1 protein and the replacement of its carboxyl portion with a sequence that encodes tuberculosis antigens. The choice of the target proteins is determined by the fact that these are expressed at different phases of the mycobacterium’s life cycle and contain well-characterised epitopes which recognise CD4+ and CD8+ T-cells for the induction of balanced cell-mediated immune responses. The immunogenic proteins include ESAT-6, TB10.4 and HspX for making the DNA and vector-based vaccines [6].

This study assessed the safety and protective efficacy of two candidate vaccines using influenza vectors expressing the ESAT -6 and TB10.4 of M. bovis in outbred Balb/c mice to protect against bovine tuberculosis.

## 2. Materials and Methods

### 2.1. Vaccine

The candidate bovine tuberculosis vaccine was administered by subcutaneous injection. The vaccine contains a reassortant recombinant type A influenza virus, the A/Puerto-Rico/8/34 H1N1 strain (a donor of PB1, PB2, PA, NP and chimeric NS genes) and the A/chicken/Astana/6/05(H5N1) strain (a donor of HA, NA and M genes) which express the ESAT-6 and TB10.4 mycobacterial antigens of the M. bovis-8 strain [internal reference code: FLU NS_ESAT 6 and FLU NS_TB10.4]. The recombinant viruses were obtained with standard reverse genetics techniques using eight pHW2000 bidirectional plasmids [7]. The obtained virus was cultivated on 10–11-day-old RCE chicken embryos free from specific pathogens at 37 ± 0.5 °C. After incubation for 48 h, the embryos were cooled to 2–8 °C. The allantoic fluid was collected, clarified by centrifugation at 9000× *g* for 30 min, and mixed in a 1:1 ratio with a sterile stabilising medium containing 12% peptone (final concentration 6%) and 6% sucrose (final concentration 3%).

The study involved testing three experimental batches of each of the two versions of the candidate bovine tuberculosis vaccine:

- Three FLU NS_ESAT 6 and TB10.4 pilot batches containing 15% Montanide Gel adjuvant (Seppic, France);
- Three FLU NS_ESAT 6 and TB10.4 pilot batches without adjuvant.

The study used saline-treated and naive intact animal groups as negative control groups.

### 2.2. Bacterial Strain

The study used a virulent M. bovis-8 strain obtained from Research Institute for Biological Safety Problems’s (RIBSP) microbiology laboratory collection. The bacterial cells were cultured on a solid Lowenstein-Jensen medium (Remel Inc., Lenexa, KS, USA) at 37 ± 0.5 °C. All experiments with live cells were conducted in a Biosafety Level 3 (BSL-3) laboratory.

### 2.3. Animal Studies

**Figure 1:**
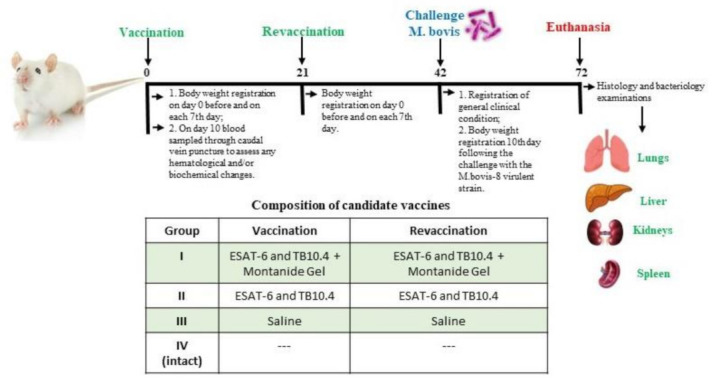
Study timeline and sampling schedule. Balb/c (8 animals per group) were immunised with two subcutaneous injections 21 days apart. G.1 was vaccinated with a vaccine consisting of an influenza vector expressing ESAT-6 and TB10.4 proteins with the addition of Montanide gel. G.2 was vaccinated with a vaccine consisting of an influenza vector expressing ESAT-6 and TB10.4 proteins. The control group (G.3) was immunised with saline. 21 days after revaccination, mice were subcutaneously infected with a virulent M. bovis-8 strain. M. bovis-8 infection was monitored over a 30-day observation period. 30 days after the challenge, euthanasia was performed, followed by a bacteriological and histological examination.

The study used 64 outbred white mice aged 8–10 weeks (n = 8 per group) with an average weight of 18–25 g. The animals had been quarantined for 15 days before the experiment. All experimental designs are given in Figure 1.

Before the beginning of the experiment, mice were tested for allergic reactions by an intracutaneous tuberculin test to exclude those sensitised to M. bovis mycobacteria. In addition, the animals received specialised feed twice daily, and had unlimited access to drinking water. At the time of the challenge, animals were kept in a special isolated cage to prevent the spread of the wild-type virus into the environment.

Mice were divided into groups by randomisation. The randomisation criteria included lack of external signs of disease and homogenous weight (± 20%) across the groups.

The study was conducted by national and international animal handling laws and recommendations. In addition, the study protocol was approved by RIBSP’s institutional ethics committee (Protocol #10, dated 28 September 2020).

### 2.4. Safety Studies

For safety studies, groups of eight 8–10-week-old mice were immunised twice by the subcutaneous introduction of lg 6.25 EID_50_/1 mL of one of the two versions of the candidate vaccine per animal. The two vaccinations were carried out 21 days apart.

Mice’s clinical condition was observed throughout the whole duration of the 72-day experiment. This included documenting the animals’ general condition, such as feed and water consumption; the state of skin, hair and mucous membranes; behavioural responses; and motor activity (Figure 1). The mice’s body weight was recorded on day 0 before and on each 7th day after vaccination and re-vaccination, as well as on each 10th day following the challenge with the M. bovis-8 virulent strain (Figure 1).

On the 10th day after the first vaccination, the animals sampled their blood through caudal vein puncture to assess any hematological and biochemical changes (Figure 1) [8,9,10].

G.3 and G.4 are compared in this study to determine the possible effect/stress of needle insertion during immunisation. Mice of G.3, treated with saline, were used to analyse the body’s general condition and biometrics after needle insertion. Mice of G.4 to determine the general state of animals under normal detention conditions, without any stressful situations.

### 2.5. Protection Studies

To evaluate protection, mice were challenged with virulent strain M. bovis-8 subcutaneously with 1 × 10^6^ CFU/mouse in 0.2 mL of saline on the right side of the groin. The protective efficacy was assessed 30 days after infection by determining the bacterial plating in the lungs, calculating the protection index and performing a histological assessment of the inflammatory process in the lungs, liver, kidney and spleen.

### 2.6. Blood Test

Blood samples were collected from the caudal vein and transferred to tubes with and without ethylenediamine tetraacetic acid (EDTA) for further hematology tests in a Sysmex XN-1000 (Sysmex, Landskrona, Sweden) automatic analyser to examine hemoglobin, hematocrit, red blood cells, eosinophils, monocytes and lymphocytes.

Biochemical tests of blood serum samples were used to examine nine parameters as follows: total protein; urea; creatinine; glucose; total cholesterol; total bilirubin; conjugated bilirubin; aspartate aminotransferase (AST); and alanine aminotransferase (ALT) (reagents from BioSystems, Barcelona, Spain), using an A25 BioSystems analyser.

### 2.7. Bacteriology

For bacteriology tests, serial dilutions of homogenised tissue of both lung samples were cultured on Lowenstein-Jensen solid media (Remel Inc., Lenexa, KS, USA). The acid-fast of the isolated mycobacteria was confirmed by Ziehl-Neelsen staining. The method’s sensitivity threshold was 2 × 10^3^ colony forming units (CFU). M.bovis growth was expressed in a decimal log (lg) based on the number of CFU per lung weight. The organ’s protection index was calculated by subtracting the CFU lg of immunized mice from that of the saline recipient control group. In the analysis of results, the protection index of ≥0.5 lg was considered an indicator of a positive mycobacterial growth inhibition effect.

### 2.8. Histology Tests

Thirty days after the challenge, mice were euthanised by introducing 10% chloral hydrate (Acros Organics, Geel, Belgium) intraperitoneally. A visual macroscopy examination of internal organs for the presence of lesions was performed after the necropsy. Whole lungs, liver, kidney and spleen were fixed in a 10% neutral formalin (Sigma, Taufkirchen, Germany) for histology examination (Figure 1). Histologic sections were prepared using a hematoxylin and eosin solution (Sigma, Taufkirchen, Germany).

### 2.9. Statistical Analyses

The average values of the studied parameters, the standard errors and the significance of the difference between results were determined using the GraphPad Prism 8 statistics software (GraphPad Software, Inc., La Jolla, CA, USA). Body weight values were expressed as the mean ± standard deviation (SD). The significance of the differences in survival was evaluated using the Log-Rank Test. *p* < 0.05 was considered statistically significant.

## 3. Results

No deviations in the animals’ somatic or neurological status were detected while observing the experimental and control groups. The animals appeared healthy and responded appropriately to tactile, sound and light stimuli; their hair coat was shiny, even and smooth. The growth of body weight in experimental group animals starting on day 7 of the experiment and following re-vaccination was comparable with that of the G.3 and G.4 animals. These data are significant, as the tested vaccine does not adversely impact laboratory animals’ body weight.

Under normal physiological conditions, warm-blooded animals maintain constancy of their blood’s morphological and chemical composition and physical and chemical properties. Changes in the blood composition, which often result from disrupting the physiological activity of various systems or organs by drugs and immunobiological preparations, may impact the body’s normal functioning. The study used laboratory instrumental techniques to perform a complete blood count (measuring hemoglobin, hematocrit, red blood cells, neutrophils, basophils, eosinophils, lymphocytes and monocytes) on the 10th day after the initial vaccination. Results of hematology tests performed on peripheral blood samples to assess the candidate vaccines are shown in Table 1 below.

Data in Table 1 demonstrate no significant differences in hemoglobin and hematocrit levels between the control and experimental groups. Moderate growth of nuclear rod cells and monocytes can be observed in vaccinated mice, which can be interpreted as resulting from the immune response’s activation. A slight drop in eosinophil count in vaccinated animals indicates the absence of allergic reaction in the mice to the candidate vaccine.

To provide a thorough assessment of changes in the physiological state of macroorganisms caused by the candidate vaccine, the study examined the most informative indicators of critical metabolic enzymes in the mice’s blood sera, as well as the parameters of protein, carbohydrate and lipid metabolism—the total protein, urea, creatinine, cholesterol, glucose, total and conjugated bilirubin, AST and ALT (Table 2).

Biochemistry tests did not detect any difference in total protein between the vaccinated and control groups (G.3 and G.4), even with each experimental batch of the vaccine administered. However, hyperproteinemia was a steady trend in each of the four groups. An analysis of the literature shows that the values obtained are the norm in such experiments [11].

**Figure 2:**
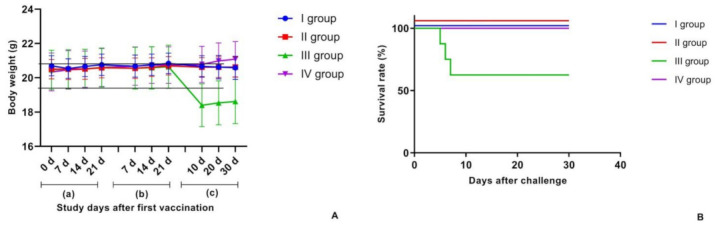
Biometric analysis during safety testing in mice, weight dynamics. (**A**) Dynamics of changes in body weight of mice (a) after the first vaccination, (b) after the revaccination and (c) after the challenge with a virulent M. bovis-8. (**B**). Registration of survival after the challenge with a virulent M. bovis-8. The entire observation period was 72 days. The standard deviations (SD) for the mean per cent deviation of body weight values of the groups are presented as error bars. In addition, survival curves were analysed for the experimental and control groups using LogRank Test criteria with statistical significance at *p* = 0.007.

The study also examined the survival rate in mice challenged with M. bovis-8. Natural deaths were recorded from day one to day thirty of the experiment; mortality curves for each group are shown in Figure 2B.

Observation over 21 days following the initial vaccination showed an increase in the animals’ body weight in each group. Towards the end of the observation period, following re-vaccination, the experimental group animals’ body weight grew by 0.67–0.87%, while the intact group animals gained 1.96%. None of the experimental groups differed significantly regarding the average body weight of the control group animals that had received saline (*p* > 0.5).

After the second vaccination, on day 42 of the experiment, mice in all groups except G.4 were challenged with a virulent M. bovis-8 strain to assess the protective activity of the candidate vaccine by histology and by culturing M. bovis from the lung tissue after euthanasia.

The animals’ behavioural patterns were observed over 30 days from the day they had been challenged with mycobacteria to the day they were euthanised. While experimental group animals demonstrated the candidate vaccines’ protective effect, the G.3 lost a mouse on days 5, 6 and 7 (Figure 2B). Before death, animals manifested hypodynamia, tachypnea, loss of appetite and an unkempt coat. A post-mortem examination of dead mice established signs of acute congested hyperemia of the lungs, the subcutaneous tissue, the liver and the kidneys, which is a characteristic sign of acute infection.

The G.3 mice manifested coat changes, poor appetite and loss of activity. On the contrary, vaccinated mice lost their appetite upon vaccination, but restored it two days later and started gaining weight afterwards. The M. bovis-8 load in the lungs and the protection index of the mice are shown in Table 3.

A specific reduction in M. bovis-8 growth was also observed in group 1 (*p* < 0.05); however, the protection index in this group was relatively low (+0.2 lg). G.2 showed a reduced level of M. bovis-8 (*p* < 0.01) isolation on the one hand, and a somewhat high protection index on the other (+0.62 lg). All these demonstrate the candidate vaccines’ prophylactic effect. Accordingly, as seen from the table data, group 3 had M. bovis-8 growth but no protection index.

**Figure 3:**
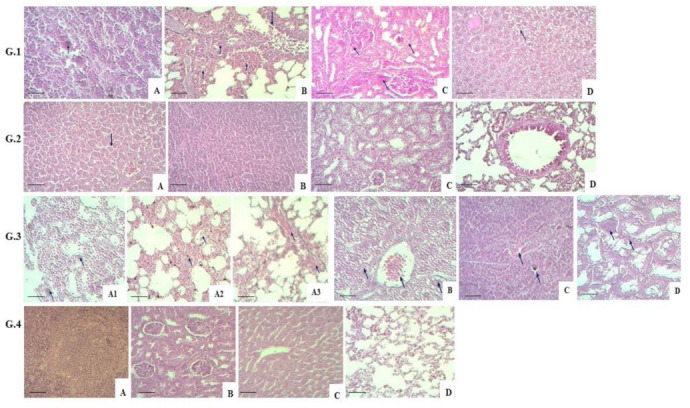
Histological structure of studied mice groups. **G.1.** (scale bar = 450 µm, 400× magn.) **A.** Lymphocytes and macrophages in the spleen; **B.** Peribronchial inflammation with bronchial epithelium desquamation and interalveolar inflammation with macrophages in the alveolar lumen; **C.** Interstitial inflammation with a macrophage and renal parenchymal dystrophy; and **D.** Parenchymal dystrophy and necrosis of hepatocytes in the liver. **G.2.** (scale bar = 800 µm, 200× magn.) **A.** Vascular congestion and hepatic tissue edema; **B.** Focal spleen edema; **C.** Renal structure restoration; and **D.** Lung structure restoration. **G.3.** (scale bar = 800 µm, 200× magn.) **A1.** Macrophage response in lung tissue; **A2.** Serous and hemorrhagic exudate in lung tissue; and **A3.** Vascular congestion in lung tissue; (scale bar = 450 µm, 400× magn.) **B.** Vascular congestion, dystrophy and necrotic hepatocytes in the; **C.** Vascular congestion, presence of mononuclear macrophages in the spleen; and **D.** Tubular epithelium dystrophy and multinucleate macrophages in kidneys. **G.4.** Regular histological structure (scale bar = 800 µm, 200× magn.) **A.** Spleen; **B.** Kidneys; **C.** Liver; and **D.** Lungs. Arrows indicate representative changes.

A histology examination of G.1 mice indicated a specific change in the spleen’s structure due to the shrinkage of lymphoid follicles, which contained large mononuclear and multinucleated cells. Morphological changes observed in the lungs were characterised by a productive peribronchial inflammation of focal nature with vascular congestion. The mice had a desquamated bronchial epithelium. Other parts of the lungs contained a thickened interalveolar septum resulting from a productive inflammation with macrophages. The interstitial renal tissue contained inflammation with isolated macrophages. The tubular epithelium was affected by parenchymal dystrophy. The liver’s histological structure was broken by focal hepatocyte parenchymal dystrophy. Additionally, necrotic liver cells were observed in several parts of the liver (G.1.A–D in Figure 3). 

Histology examination of G.2 mice indicates no significant changes in liver tissues. There are congested blood vessels and dilated hepatic sinusoids due to edema. The spleen has a typical tissue structure, and preserves the red and white pulp with lymphoid follicles; focal tissue oedema is present. The kidneys keep their structure; parenchymal dystrophy is visible in isolated tubular epithelium cells. The lungs ultimately preserve their typical histological structure. No changes are seen in the bronchial area histological structure (G.2.A–D in Figure 3). 

Histology examination of G.3 animals’ tissues indicates no changes in the lung tissue structure. The thickened interalveolar septa and alveolar lumens have large mononuclear and multinucleated cells—the macrophages. Other parts of the lungs, the interalveolar septa and alveolar lumens, contain focal serous and hemorrhagic exudate—the studied lung part feature impaired circulation through vascular congestion (G.3.A1–A3 in Figure 3). The liver preserves its histological structure; however, some parts of the liver contain hepatocytes with foamy cytoplasm and blurred nucleus contours. Some cells do not have a nucleus, and are seen as optically empty pink masses. The central vein is plethoric. A microscopy examination indicates a preserved histological structure of the spleen, though with shrunken lymphoid follicles. Large mononuclear cells and vascular congestion are present. The renal structure is changed due to the dystrophy of tubular epithelium. The tubular epithelium cells contain vacuoles filled with cytoplasmatic fluid. The cells’ nuclei cannot be delineated clearly. Large multinucleated cells are present (G.3.B–D in Figure 3).

The histological structure of G.4 mice’s organ tissues was used to compare changes in the three other groups. A healthy spleen structure is characterised by a readily distinguishable red and white pulp represented by lymphoid follicles. The kidneys have a structure with an unchanged cortex and medulla. The liver preserves its histological structure with the central vein and radiating hepatic cords. In G.4 mice, the lung tissue also has a regular histological structure with unchanged alveoli, bronchi of various calibres and vessels (G.4.A–D in Figure 3). 

Thus, comparing organ (liver, spleen, kidneys and lungs) tissues in experimental and control group animals indicated significant morphological differences after the M. bovis-8 challenge. The lung tissues of groups 1, 2 and 3 animals developed productive inflammation with macrophage response against impaired blood circulation. In the other organs, the mycobacteria’s virulence caused damage in the form of parenchymal dystrophy and microfocal necrosis. In addition to these, isolated macrophages were present.

A double immunisation with the adjuvant-free version of the candidate vector vaccine for bovine tuberculosis (G.2) led to less marked changes and enhanced restoration processes in mice’s organ tissues, demonstrating a robust protective action of the candidate vaccine.

## 4. Discussion

The primary approach toward controlling the spread of tuberculosis is provided by prophylactic vaccination.

In Commonwealth of Independent States (CIS) countries, the vaccination of cattle against bovine tuberculosis is limited to the use of Bacille Calmette-Guérin (BCG). Finding safe and effective means of immunisation for this disease remains a pressing issue in developing countries, where tuberculosis is prevalent in domestic and wild animals. BCG is currently the yardstick for judging all other vaccines, and two general ways can be used to develop vaccines that will have more robust protection against TB in cattle than BCG. One way is to increase BCG’s defence through complementary or booster vaccines. The other way is to develop a vaccine that completely replaces BCG. One of the most effective vaccination examples against bovine tuberculosis is based on priming the immune system with BCG and then boosting it with a subunit vaccine containing the protective antigens present in BCG (heterologous booster strategy). Subunits are based on DNA or viral vector booster vaccines. A variant of this theme is the simultaneous vaccination of BCG and subunit vaccines [12,13,14].

To reduce the response to the tuberculin skin test and create a safer vaccine for people with weakened immune functions, BCG has been modified by inactivating or changing some of its genes. An auxotrophic mutant of BCG has been produced, which has mutations in genes involved in the metabolism of leucine and methionine. These mutants can no longer grow on a minimal medium, and only grow when the appropriate amino acids are added. Their ability to grow in the body is also reduced. Vaccination of mice with these auxotrophic mutants has induced resistance to *M. tuberculosis* infection. Cattle vaccinated with the BCG leucine auxotrophic mutant will not cause a skin reaction to bovine PPD; however, the protective effect on bovine tuberculosis has not been evaluated.

According to the World Organisation for Animal Health (OIE), Kazakhstan is officially considered free from bovine tuberculosis. However, vaccination remains relevant given the zoonotic nature of M. bovis [15]. Several M. bovis candidate vaccines have been tested on laboratory animals, including modified versions of BCG, DNA vaccines and other vaccines that use M. bovis immunodominant proteins [16,17,18,19,20].

One of the most consistent and successful ways to improve the effectiveness of BCG against experimental challenges is to boost vaccines with viral vectors. The data generated in the past eight years showed that this replication-deficient human recombinant adenovirus type 5 vaccine expressing mycobacterial antigen 85A could repeatedly improve the efficacy of BCG in vaccinated cattle when applied to the BCG main/booster vaccine program. Furthermore, compared with only BCG vaccination, the increase in the proportion of vaccinated animals with no visible tuberculosis lesions and the decrease in general pathology and histopathology demonstrate this improvement over BCG [21,22].

One way to enhance the immunogenicity of mycobacterial antigens is to use attenuated viruses or bacteria as vectors to deliver and express them. Currently, some studies are being conducted to examine the protective potential of attenuated viral (adenovirus, cowpox) and bacterial (Francisella tularensis, Shigella, Salmonella typhimurium) vectors carrying protective mycobacterium tuberculosis antigens (ESAT6, 85A, SOD A, etc.). One of the main requirements for developing vector vaccines is a reliable attenuation of the vector strain and its safety. At the same time, recombinant virus replication must be accompanied by a sufficient production of the inserted protein to shape the immunisation’s protective effect [23,24].

Considering the promising results of live viral vectors, an alternative strategy for developing safe and effective vaccines for infectious diseases is using genetically modified vectors, i.e., non-pathogenic microorganisms (bacteria and viruses) that express the agent’s antigens [25,26]. The main challenge in building vector vaccines is to select an optimal and safe vector and insert genes that will protect the recipient [27]. When developing tuberculosis vaccines, particular importance should be attached to their safety and effectiveness. To assess these, our study used outbred white mice to conduct experiments involving two (subcutaneous) immunisations with two versions of the tested vaccine 21 days apart. In addition, the vaccine’s protective efficacy in mice was assessed by challenging (subcutaneously) with a virulent M. bovis-8 strain on day 22 after re-vaccination. The choice of outbred white mice was based on the fact that these animals are often used in human tuberculosis vaccine studies. Furthermore, laboratory mice are widely utilised because of their affordable price [28,29].

Similar studies assessing the safety of recombinant influenza viruses expressing M. tuberculosis’ proteins ESAT-6 and Ag-85 in laboratory animals such as mice and guinea pigs have proved them to be completely safe. They have also demonstrated that animals immunised with recombinant influenza viruses developed a T-cell immune response and a level of protection that was not inferior to those induced by commercial BCG vaccines [30,31].

The genomes of M. bovis and M. tuberculosis are more than 99.95% identical at the nucleotide level [32]. Previous studies identified mycobacterial proteins, among which the secretory ESAT-6 and TB10.4 of M. bovis have potential antigenic properties [33,34,35]. A group of known antigens affected by deletions from M. bovis makes up the ESAT-6 family. The ESAT-6 protein was initially described as a potent T-cell antigen secreted by M. tuberculosis [36]. Considering these properties, the TB10.4 and ESAT-6 proteins provide prospective antigens to develop candidate vaccines for bovine tuberculosis [35].

This study involves two subcutaneous vaccinations of white mice 21 days apart to assess the safety and effectiveness of the candidate vaccine to inform further studies on guinea pigs and cattle. To this end, the study uses the NS gene of the avian influenza type A Puerto-Rico/8/34 (H1N1) strain. The Puerto-Rico/8/34 (H1N1) strain can effectively replicate in mice, initiate the infectious process and bring about disease symptoms.

Both candidate vaccine versions were proven safe based on biometric assessment and analysis of immunised mice’s blood composition. Throughout the observation period, no behavioural changes, deaths or drops in body weight were reported in vaccinated mice. The vaccination did not cause significant differences in the experimental and control group animals’ morphological composition of peripheral blood hemoglobin and hematocrit. A slight reduction in eosinophils was observed, however, which demonstrates an absence of the allergising effect of the vaccine on the mice. The changes in the blood’s biochemical composition can be interpreted as caused by immune response activation.

The study established the candidate vaccine’s protective effectiveness through bacteriology and histology examinations of mice challenged with a virulent M. bovis-8 strain. Homogenised lung tissues of mice immunised with the adjuvant-free version (G.2) produced a low number of mycobacteria CFU in the culture. The number of CFU corresponded to a low isolation rate of M. bovis-8 (*p* < 0.01); additionally, this group had a relatively high protection index (+0.62 lg). A limitation of the study is the lack of normalisation of lung weights in the CFU data. A similar result was obtained through histology examination, where group 2 manifested less marked changes in organ tissues on the one hand and enhanced restoration processes on the other, both of which demonstrate a robust protective effect of the relevant candidate vaccine version.

There was no comparative evaluation with the BCG vaccine, since this work aims to determine the safety of the new vector vaccine. The following studies will compare its efficacy and protective efficacy with challenging virulent M. bovis strain in susceptible animals (guinea pigs and cattle) to bovine tuberculosis with the commercial BCG vaccine.

The study’s results provide evidence that the tested candidate vaccine using the NS1 influenza vector expressing ESAT-6 and TB10.4 (M. bovis protective antigens) is safe and effective for use on mice, and can therefore inform further studies investigating the immunogenicity and protective properties on animals susceptible to the M. bovis-8 tuberculosis (guinea pigs and cattle).

## 5. Conclusions

The study’s results have demonstrated the safety and protective effectiveness of a new bovine tuberculosis candidate vaccine that uses a recombinant influenza vector expressing M. bovis proteins ESAT-6 and TB10.4 from the open reading frame of the NS gene applied in two rounds (subcutaneously). In addition, the data obtained allow the recommendation of the candidate vaccine for further studies to assess its immunogenicity and protection against M. bovis-8 tuberculosis in susceptible animals such as guinea pigs and cattle.

## Figures and Tables

**Table 1:** Candidate vaccine’s impact on the morphological composition of outbred white mice’s peripheral blood.

Blood Parameters Studied	Study Groups			
	I	II	III	IV (Intact Group)
Hemoglobin, g/dL	40.00 ± 1.27	40.00 ± 6.66	40.00 ± 0.68	41.57 ± 1.23
Hematocrit, %	0.32 ± 0.03	0.38 ± 0.02	0.39 ± 0.02	0.34 ± 0.04
Red blood cells, ×10^7^ L	4.0 ± 0.3	4.1 ±0.5	3.1 ± 0.2	3.4 ± 0.3
Eosinophils, %	1.2 ± 0.0	1.4 ± 0.1	1.5 ± 0.2	1.5 ± 0.3
Monocytes, %	7.7 ± 1.2	5.9 ± 1.2	5.3 ± 1.2	6.7 ± 1.2
Lymphocytes, %	47.3 ± 3.3	45.7 ± 1.3	42.7 ± 3.7	45.0 ± 4.0

**Table 2:** Impact of tested candidate vaccine on critical biochemical parameters of peripheral blood in outbred white mice.

Blood Parameters Studied	Study Groups			
	I	II	III	IV (Intact Group)
Total protein, g/L	58.9 ± 4.4	48.0 ± 1.4	40.1 ± 5.7	52.1 ± 1.2
Urea, mmol/L	3.8 ± 0.1	3.6 ± 0.8	4.2 ± 1.4	5.6 ± 1.1
Creatinine, umol/L	52.3 ± 3.5	48.4 ± 4.1	39.9 ± 5.1	48.5 ± 2.3
Glucose, mol/L	5.0 ± 1.7	5.0 ± 1.9	5.5 ± 1.4	8.2 ± 2.2
Cholesterol, total, mmol/L	1.0 ± 0.8	1.0 ± 0.4	1.4 ± 0.3	0.7 ± 0.2
Bilirubin, total, mmol/L	0.004 ± 0.001	0.008 ± 0.001	0.008 ± 0.002	0.010 ± 0.004
Bilirubin, conjugated, mmol/L	0.003 ± 0.000	0.004 ± 0.000	0.003 ± 0.000	0.006 ± 0.001
AST, mmol/L-s	1.56 ± 0.13	1.25 ± 0.06	1.45 ± 0.32	1.92 ± 0.05
ALT, mmol/L-s	0.04 ± 0.01	0.07 ± 0.01	0.05 ± 0.01	0.31 ± 0.03

**Table 3:** Isolation of mycobacteria from lungs of mice challenged with M. bovis-8.

Groups	Experiment Conditions	Log_10_ of the Number of Viable Bacteria in the Lungs	Protection Index (Log_10_)
I	ESAT-6 and TB10.4 + Montanide Gel	2.39 ± 0.182	+0.2
II	ESAT-6 and TB10.4	1.97 ± 0.447	+0.62
III	Saline	4.89 ± 0.044	-

## Data Availability

Not applicable.
